# Microvascular Dysfunction, Mitochondrial Reprogramming, and Inflammasome Activation as Critical Regulators of Ischemic Stroke Severity Induced by Chronic Exposure to Prescription Opioids

**DOI:** 10.1523/JNEUROSCI.0614-24.2024

**Published:** 2025-01-03

**Authors:** Enze Sun, Silvia Torices, Olivia M. Osborne, Michal Toborek

**Affiliations:** Department of Biochemistry and Molecular Biology, University of Miami Miller School of Medicine, Miami, Florida 33136

**Keywords:** blood–brain barrier, inflammasome, ischemic stroke, mitochondria, prescription opioids

## Abstract

The opioid epidemic endangers not only public health but also social and economic welfare. Growing clinical evidence indicates that chronic use of prescription opioids may contribute to an elevated risk of ischemic stroke and negatively impact poststroke recovery. In addition, NLRP3 inflammasome activation has been related to several cerebrovascular diseases, including ischemic stroke. Interestingly, an increase in NLRP3 inflammasome activation has also been reported in chronic opioid exposure. Given the pivotal roles of the blood–brain barrier (BBB) and oxidative stress in ischemic stroke pathophysiology, this study focuses on the impact of chronic exposure to prescription opioids on the integrity of cerebrovascular microvasculature, endothelial mitochondrial homeostasis, and the outcomes of ischemic stroke in male wild-type and NLRP3-deficient mice. Our results demonstrate that chronic opioid exposure can compromise the integrity of the BBB and elevate the generation of reactive oxygen species (ROS), resulting in endothelial mitochondrial dysfunction and apoptosis activation. We also provide evidence that opioid exposure enhances inflammasome activation and inflammatory responses and increases the severity of an ischemic stroke. The antioxidant *N*-acetylcysteine ameliorated these opioid-induced alterations and accelerated the poststroke tissue restoration and functional recovery processes in opioid-exposed mice. Importantly, there was also a significant decrease in ischemic stroke damage in the NLRP3-deficient mice with chronic opioid exposure as compared with wild-type controls. These findings indicate that chronic exposure to prescription opioids impacts the outcome of ischemic stroke by damaging microvascular cerebral integrity through inflammasome activation and mitochondrial dysfunction.

## Significance Statement

Misuse of opioids has become one of the most important public health problems. Growing evidence indicates that chronic use of prescription opioids may contribute to an elevated risk of ischemic stroke and negatively impact poststroke recovery. In the present study, we hypothesize that microvascular dysfunction can underlie the impact of prescription opioids on an ischemic stroke. Our novel results demonstrate that opioid exposure leads to mitochondrial dysfunction in the brain microvascular endothelium, compromised blood–brain barrier integrity, enhanced inflammatory responses, and more severe effects of an ischemic stroke. Importantly, the NLRP3 inflammasome-deficient mice or treatment with *N*-acetylcysteine attenuated these alterations and enhanced poststroke tissue and functional recovery, providing valuable therapeutic options for people with opioid use disorder.

## Introduction

There has been a dramatic increase in the use of prescription opioids for the treatment of chronic, noncancer pain in recent years (Tobore, 2021; Paice et al., 2023). An unprecedented epidemic of the misuse and abuse of opioids has become one of the most consequential public health problems in the USA, which is best reflected by an alarming increase in opioid overdose deaths (Baldwin et al., 2021). Besides the serious risks of addiction, abuse, and overdose, emerging clinical data indicates that intense or long-term use of opioids is linked to a higher risk of developing cerebrovascular diseases, such as stroke. Indeed, the percentage of stroke occurrences among hospitalized patients has significantly increased along with the combination of opioid abuse (Omran et al., 2019).

The brain microvascular endothelium has been shown to be a target of opioid toxicity. Morphine, a typical prescription opioid, can induce the breakdown of the blood–brain barrier (BBB) by disrupting the expression of tight junction (TJ) proteins in endothelial cells (H. C. Liu et al., 2004; Mahajan et al., 2008), which are critical for the maintenance of brain homeostasis. Increased BBB permeability can lead to uncontrolled trafficking of immune cells into the brain parenchyma, resulting in inflammasome activation and neuroinflammation, one of the major contributors to vascular damage and mortality in ischemic stroke (Hu et al., 2023; Li et al., 2023).

An inflammasome is an intracellular multiprotein complex of receptors and sensors that are a part of the innate immune system (Broz and Dixit, 2016). Multiple inflammasome proteins have been related to stroke severity (Fann et al., 2013; Kim et al., 2020). Among them, the NOD-like receptor protein 3 (NLRP3) inflammasome plays a crucial role in brain injury recovery after an ischemic stroke (Torices et al., 2023). The NLRP3 inflammasome is formed by the NLRP3 protein engaging with the adapter protein ASC (apoptosis-associated speck-like protein containing CARD) and the inflammatory pro-caspase-1. When activated, it induces pro-IL-1β and IL-18 transformation into their active forms, promoting proinflammatory responses. It has been shown that inhibition of NLRP3 inflammasome activation significantly reduces BBB damage, ischemic stroke infarct size, and endothelial cell death through pyroptosis regulation (Ismael et al., 2018).

Chronic opioid use has been shown to result in analgesic tolerance and hyperalgesia, in which oxidative stress and generation of reactive oxygen species (ROS) play a critical pathological role (Tobore, 2021; Newman et al., 2022). ROS production and reactive nitrogen species are the key targets in ischemic stroke outcomes by regulating BBB damage (Chen et al., 2020) and inflammasome activation (Minutoli et al., 2016). Accumulated in vitro and in vivo evidence indicates that opioid exposure can modulate the antioxidant response in brain endothelial cells (Reymond et al., 2022) and lead to robust ROS production and mitochondrial dysfunction, which can result in apoptosis and further contribute to the development of an ischemic stroke (J. L. Yang et al., 2018). While the association between chronic exposure to prescription opioids and the increased risk of ischemic stroke has been suggested in clinical reports, the mechanisms of how opioid exposure affects the outcome of ischemic stroke are still not clearly understood.

In the present study, we hypothesize that microvascular dysfunction can underlie the impact of prescription opioids on an ischemic stroke. Our results demonstrate that opioid exposure leads to mitochondrial dysfunction in the brain microvascular endothelium, compromised BBB integrity, enhanced inflammatory responses, and more severe effects of an ischemic stroke. Importantly, the NLRP3 inflammasome-deficient mice or treatment with *N*-acetylcysteine (NAC) attenuated these opioid-induced alterations and enhanced poststroke tissue and functional recovery.

## Materials and Methods

### Animals and drug treatment regimen

Male C57BL/6J and NLRP3-deficient mice (NLRP3^−/−^; The Jackson Laboratory) at 13 weeks of age were allowed to acclimate to the animal facility for 1 week with free access to food and water. All animal procedures were approved by the University of Miami Institutional Animal Care and Use Committee in accordance with National Institutes of Health guidelines and performed following the relevant guidelines and regulations. Mice were weight-matched and randomly assigned to different treatment groups. Mice were injected intraperitoneally (i.p.) with morphine sulfate (National Institute on Drug Abuse) or oxycodone chloride (Sigma-Aldrich) dissolved in saline twice per day at 6 h intervals with a daily escalating dose regimen for 2 weeks. The escalating dose range for morphine was 1–15 mg/kg, and the escalating dose range for oxycodone was 0.6–10 mg/kg, using a daily stepwise increase. The dosages were based on the extrapolation from the clinical dosages in humans (Nair and Jacob, 2016). Control mice were injected with saline as a vehicle. *N*-acetylcysteine (NAC; Sigma-Aldrich, catalog #A7250) was prepared in saline and 150 mg/kg body weight of NAC or saline was administered in conjunction with the opioid via intraperitoneal injection during the last 5 d of the treatment period.

### Middle cerebral artery occlusion surgery

Ischemic stroke was induced by the middle cerebral artery occlusion (MCAO) surgery as previously described (Bertrand et al., 2017) by inserting a silicone-coated suture into the common cerebral artery and blocking blood flow to the middle cerebral artery for 60 min. Afterward, the suture was removed, and blood flow was restored. Brains were harvested at 24 h postreperfusion, sliced with a 1 mm brain matrix, and stained with 2,3,5-triphenyltetrazolium chloride (Sigma-Aldrich, catalog #T8877-10G). The images were captured using a digital camera (Nikon), and the infarct volume was analyzed using ImageJ software (Bertrand et al., 2019).

### Isolation of brain microvessels

Deeply anesthetized mice were perfused with saline, after which brains were harvested and brain capillaries were isolated as described previously (Park et al., 2016). Briefly, brain tissue was homogenized in a cold isolation buffer (102 mM NaCl, 4.7 mM KCl, 2.5 mM CaCl_2_, 1.2 mM KH_2_PO_4_, 1.2 mM MgSO_4_, 15 mM HEPES, 25 mM NaHCO_3_, 10 mM glucose, 1 mM sodium pyruvate) and supplemented with proteinase inhibitors (Santa Cruz Biotechnology, catalog #sc-24948a). Then, 26% dextran (MW, 150 kDa) in isolation buffer was added, mixed, and centrifuged (5,800 × *g*; 4°C) for 20 min. The pellets were resuspended with cold isolation buffer and filtered through a 300 μm (Thermo Fisher Scientific, catalog #NC1480938) and a 120 μm (Merck, catalog #NY2H09000) nylon mesh filter successively. Filtered homogenates were recentrifuged (1,500 × *g*; 4°C) for 10 min and resuspended in 200 μl of the isolation buffer.

### Endothelial cell culture and treatment

Primary human brain microvascular endothelial cells (hBMEC, Cell Systems, catalog #ACBRI 376) were cultured in a complete classic medium with serum and CultureBoost (Cell Systems, catalog #4Z0-500). Cells were exposed to 50 μM morphine sulfate or oxycodone chloride twice per day for 2 d and then were lysed or fixed for downstream characterization.

### Permeability assays

The in vivo BBB permeability assay was performed using sodium fluorescein (NaF) as described before (Bertrand et al., 2016). Briefly, mice were injected intraperitoneally with 200 μl of 10% NaF solution (Sigma-Aldrich, catalog #F6377-100G), which was allowed to circulate for 20 min. Mice were then anesthetized, blood was collected via heart puncture, and animals were perfused using normal saline. Brain hemispheres were thoroughly homogenized, cleared of debris by centrifugation, and precipitated with trichloroacetic acid (Sigma-Aldrich, catalog #T6399), followed by centrifugation. Supernatants were mixed with 0.05 M sodium tetraborate buffer, and the fluorescence was measured using a fluorescent plate reader (485 nm Ex; 525 nm, Em). In addition to the brain, plasma NaF levels were also assessed to control injection variation.

In vitro endothelial permeability assays were performed in 12-well 0.4 μm Transwell plates (Sigma-Aldrich, catalog #CLS3397). A total of 5 × 10^4^ hBMEC cells were seeded per insert. The medium was changed every 48 h, and the monolayer formation was monitored using transepithelial electrical resistance. Five days after seeding, the medium was changed in the upper chamber for opioid treatment. Following exposure, phenol red-free EBM medium (Lonza, catalog #CC-3129) containing 4 and 10 kDa fluorescein isothiocyanate (FITC)-dextran (Millipore Sigma, catalog #FD4S-250MG and catalog #FD10S-250MG, respectively) was added to the upper chamber at the concentration of 0.5 mg/mL. Fluorescent marker translocation was analyzed after 60 min of incubation by transferring 100 μl aliquots from the lower chamber to a 96-well plaque and reading the fluorescence at 485 nm (Ex) and 525 nm (Em).

### Real-time PCR

Total mRNA from the brain hemisphere was isolated using the QIAamp Lipid Tissue Mini Kit (Qiagen, catalog #74804). Total RNA was quantified using NanoDrop 2000 (Thermo Fisher Scientific), and 100 ng of mRNA was used in every reaction. Reverse transcription and qPCR reactions were performed using the qScript XLT 1-Step RT-qPCR Tough Mix (Quantabio, catalog #89236-676). The following primers were used for gene amplification: mouse CXCL1 (Mm04207460_m1) and mouse matrix metalloprotease (MMP) 9 (Mm00442991_m1). GAPDH mRNA was used for sample normalization. Quantitative real-time PCR was executed using the Applied Biosystems 7500 system (Applied Biosystems). Gene expressions were calculated using the ΔCt method, with Ct representing the cycle number at threshold.

### Protein isolation and immunoblotting

Deeply anesthetized animals were perfused with saline, after which brains were harvested and immediately frozen in liquid nitrogen. Brain samples were manually lysed in 700 μl of RIPA buffer (50 mM Tris-HCl, 150 mM NaCl, 1% NP-40, 0.5% sodium deoxycholate, and 0.1% SDS, pH 7.4; Millipore Sigma, catalog #R0278-50ML) containing protease inhibitors (Santa Cruz Biotechnology, catalog #sc-24948a). Then samples were homogenized using a TissueLyser LT system (Qiagen), followed by centrifugation. Protein concentration was measured in the supernatants by the BCA Protein Assay Kit (Thermo Fisher Scientific, catalog #23223). Samples were then denatured with Laemmli sample buffer, and Western blotting was performed using TGX 4–20% gradient precast gels (Bio-Rad Laboratories, catalog #5671094) and the PVDF membrane Trans-Blot Turbo Transfer System (Bio-Rad Laboratories, catalog #170-4159). Membranes were blocked in 5% (w/v) bovine serum albumin (BSA) in TBST buffer [50 mM Tris-HCl, pH 7.6; 150 mM NaCl; 0.1% (v/v) Tween 20] and incubated overnight at 4°C with the following primary antibodies (1:1,000 in 5% BSA–TBS): anti-occludin (Thermo Fisher Scientific, catalog #71-1500), anti-claudin-5 (Thermo Fisher Scientific, catalog #341600), anti-ZO-1 (Thermo Fisher Scientific, catalog #339100), anti-DRP1 (Cell Signaling Technology, catalog #14647), anti-pDRP1 (Ser 616; Cell Signaling Technology, catalog #44945), anti-OPA1 (Cell Signaling Technology, catalog #80471S), anti-Bax (Cell Signaling Technology, catalog #2772S), anti-Bcl-2 (Cell Signaling Technology, catalog #15071T), anti-CD31 (Cell Signaling Technology, catalog #77699S), anti-FGB (Cell Signaling Technology, catalog #9740S), anti-Iba1 (Cell Signaling Technology, catalog #17198S), anti-ICAM-1 (Cell Signaling Technology, catalog #4915S), anti-AMPK (Cell Signaling Technology, catalog #5831S), anti-pAMPK (Cell Signaling Technology, catalog #2535S), anticleaved PARP (Cell Signaling Technology, catalog #5625S), anti-PARP (Cell Signaling Technology, catalog #9532S), anticleaved caspase-3 (Cell Signaling Technology, catalog #9662S), anti-caspase-3 (Cell Signaling Technology, catalog #9661S), anti-eNOS (Cell Signaling Technology, catalog #9572S), anti-ASC (Cell Signaling Technology, catalog #67824), or anti-caspase-1 (Cell Signaling Technology, catalog #24232). Detection was performed with corresponding 800CW or 680RD conjugated secondary antibodies at 1:20,000 in 5% BSA–TBS (Li-Cor, catalog #926-32210, #926-68070, #926-32211, and #926-68071) followed by scanning in the Li-Cor CLX imaging system and analyzing by the Image Studio 4.0 software (Li-Cor). GAPDH antibody (Novus Biologicals, catalog #NB600–502FR or catalog #NB600-5021R) was used at 1:200,000 as a housekeeping.

### Immunofluorescence and image analysis

Fresh microvessel samples were spread across glass microscope slides (VWR, catalog #48311-703), heat-fixed for 10 min at 95°C, and then fixed with 4% PFA for 15 min at room temperature. Similarly, endothelial cultures were fixed with 4% PFA for 15 min at room temperature. Samples were washed with phosphate-buffered saline (PBS) and permeabilized with 0.1% Triton X-100 in PBS for 10 min. Nonspecific binding was blocked with the NATS solution (20% FBS and 0.5% in PBS) for an hour. Afterward, samples were incubated overnight with the following antibodies diluted at 1:100 in 3% BSA in TBS at 4°C: anti-occludin (Thermo Fisher Scientific, catalog #71-1500), anti-TOM20 antibody (Thermo Fisher Scientific, catalog #PA5-52843), anti-ZO-1 (Cell Signaling Technology, catalog #13663S), anti-claudin 5 (Cell Signaling Technology, catalog #49564S), anti-BAX (Cell Signaling Technology, catalog #41162S), or anti-pDrp1 (Cell Signaling Technology, catalog #3455S). The next day, samples were incubated with secondary antibodies (1:400 in 3% BSA in TBS) conjugated with goat anti-rabbit Alexa Fluor 488 secondary antibody (Thermo Fisher Scientific, catalog #A-11034) or goat anti-mouse Alexa Fluor 594 secondary antibody (Thermo Fisher Scientific, catalog #A-11032) for 2 h at room temperature. After washing with PBS, slides were mounted with VECTASHIELD antifade mounting medium with DAPI (Vector Laboratories, catalog #H-1500-10). Images were captured with the Olympus FLUOVIEW 1200 Laser Scanning Confocal Microscope (Olympus) using a 60× or 100× oil immersion lens.

Quantification of TJ protein distribution was conducted by ImageJ software. Microvessels with a diameter between 4 and 10 μm were selected for analysis, and at least 25 different images per sample were analyzed. Quantification of signal levels from brain microvessels was conducted as follows: *Z*-stack images were projected to a single image using the maximum intensity selection. The microvessel area was selected based on CD31 staining as a reference marker with ImageJ. The mean signal intensity was then assessed by the measurement of the total fluorescence intensity and divided by the selection area. TJ fragmentation was measured by the number of fragments with a target signal above a threshold of 25% maximal intensity divided by the reference area.

Quantification of mitochondria morphology was performed with the ImageJ plugin MiNA (Valente et al., 2017). Tom20 was used as a mitochondria marker, and at least 15 images of Tom20 staining per sample were analyzed. The mean length of branch and number of individuals were selected to evaluate the fragmentation of mitochondria. The colocalization was analyzed with the ImageJ plugin EzColocalization (Stauffer et al., 2018).

### Multiplex cytokine assay (ELISA)

Plasma levels of proinflammatory cytokines were measured with the mouse cytokine 23-plex assay (Bio-Rad Laboratories, catalog #M06669RDPD) following the manufacturer's instructions. Cytokine levels were quantified in pg/mL.

### Quantification of ROS production

A total of 5 × 10^4^ hBMEC cells were seeded per well in 48-well plates. After reaching 100% confluency, the media was changed to apply treatment with opioids. After the treatment, the medium was changed to HBSS buffer containing 5 µM MitoSOX red superoxide probe (Thermo Fisher Scientific, catalog #M36008), and cells were incubated at 37°C for 10 min. The plate was then washed with HBSS buffer, and fluorescence was measured using the SpectraMax iD3 reader (Molecular Devices). The protein amount quantified by the BCA assay was used for normalization.

### Seahorse mito stress test

A total of 2 × 10^4^ cells per well were seeded in the Agilent XF cell culture plate (Agilent, catalog #100777-004). After reaching 100% confluency, cultures were exposed to opioids, and mitochondrial respiration was measured by the Seahorse Cell Mito Stress Test Assay (Agilent, catalog #103015-100) using a Seahorse XF24 Analyzer (Agilent). In addition, the Seahorse mito stress test with microvessels was conducted with an adapted protocol (Sure et al., 2018). Briefly, microvessels were isolated from fresh brain tissue, and then the isolated microvessels were passed through the 30 μm nylon mesh filter to remove the cell debris and then centrifuged. The microvessels were resuspended in a warm Seahorse assay medium and seeded into the Agilent XF cell culture plate (Agilent, catalog #100777-004).

#### Behavioral testing

Behavioral analyses serve as the ultimate indicators of poststroke functional recovery. Neurodeficit scores were evaluated at 24 h and 4, 7, 10, and 14 d postischemic stroke by assessing animal conditions, behavior, and motor functions by using 13 evaluation criteria (Cuomo et al., 2007). These time points reflect the pick of acute poststroke lesion (24 h) and poststroke recovery phase (4, 7, 10, and 14 d). Testing was conducted at the same time of the day and in the same room for all groups to reduce variability. The investigators performing the test were blinded, with groups’ identities being revealed only at the end of the study.

#### Statistical analysis

Statistical analyses were concluded by Welch's test or one-way ANOVA followed by Tukey's multiple-comparisons test. The significance level was considered at *p* < 0.05. All analyses were performed using GraphPad Prism Software version 7.0.

## Results

We first evaluated the impact of chronic opioid exposure on microvascular integrity, including BBB integrity, mitochondrial dysfunction, and apoptosis, i.e., the major contributors to ischemic stroke onset. Following these experiments, we assessed the impact of chronic exposure to opioids on ischemic stroke severity.

### Chronic opioid exposure disrupts BBB integrity in vivo and in vitro

**Figure 1.**
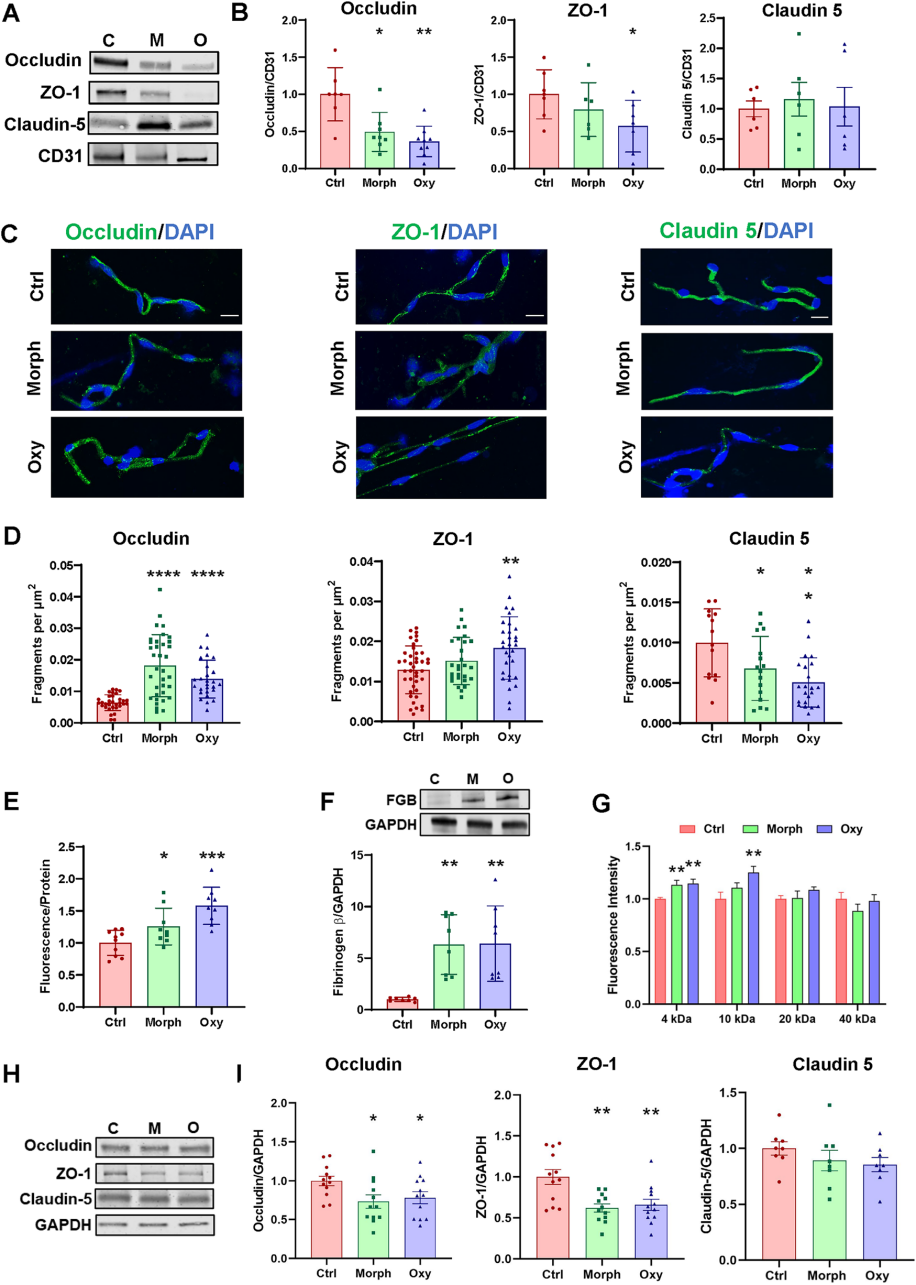
Opioid-induced disruption of microvascular barrier function. ***A–F***, Age-matched mice were injected intraperitoneally with morphine, oxycodone, or saline as control for 2 weeks, followed by brain microvessel isolation. ***A***, ***B***, The expression levels of occludin, ZO-1, and claudin-5 in microvessels were analyzed by immunoblotting. ***A***, Representative images of immunoblots. ***B***, Quantitative immunoblot results, presented as scatter plots with means ± SD. Blots were normalized to CD31, the endothelial marker (*n* = 6 animals per group, 3 independent experiments). ***C***, Representative images of microvessels immunostained in green for occludin, ZO-1, and claudin-5. The nuclei were stained with DAPI (blue). Scale bar, 20 μm. ***D***, Quantitative fragmentation results of fluorescence signal presented as scatter plots with means ± SD (*n* = 10 animals per group, 3 independent experiments). ***E***, Brain NaF extravasation as the marker of disruption of BBB integrity in control, morphine-exposed, and oxycodone-exposed mice (*n* = 10 animals per group, 3 independent experiments; means ± SD). ***F***, Expression level of fibrinogen β in brains as a marker of BBB integrity as analyzed by immunoblotting. GAPDH was used as a loading control. The same GAPDH band is shown in Extended Data Figure 1-1*A* since the same membrane was stained with different antibodies (*n* = 8 animals per group, 3 independent experiments; means ± SD). ***G***, ***H***, Cultured hBMEC were treated with 50 μM morphine or oxycodone twice per day for 3 d. Endothelial barrier integrity and TJ protein expression were measured by dextran fluorescence and immunoblotting, respectively. ***G***, Cells were cultured on a Transwell system with a medium containing fluorescein-tagged dextran of different molecular weights. The fluorescence intensity of fluorescein in the lower chamber of the Transwell system was measured at 1 h after adding the dye to the upper chamber (*n* = 12 per group, 3 independent experiments). ***H***, Left panel, Representative images of immunoblots. Middle and right panels, Quantitative immunoblots results of occludin, ZO-1, and claudin-5 expression levels. GAPDH was used as a loading control (*n* = 12 per group, 3 independent experiments). Extended Data Figure 1-1 further supports Figure 1 by providing results on inflammatory responses beyond brain microvasculature. Data are means ± SEM. *p < 0.05, **p < 0.01, ***p < 0.001, or ****p < 0.0001.

In the initial series of experiments, we evaluated the status of TJ in the brain microvessels after chronic opioid exposure. The brain tissue and blood were collected 24 h after the last dose of opioids was administered. The expression levels of three major TJ proteins, occludin, ZO-1, and claudin-5, were assessed by immunoblotting and normalized to the endothelial cell marker, CD31. The levels of occludin and ZO-1 were significantly reduced in the microvessels of both morphine- and oxycodone-exposed mice, while claudin-5 levels did not significantly change compared with the control mice (Fig. 1*A,B*). To further analyze the distribution of TJ proteins in the microvessels, all three major TJ proteins were individually immunostained, and the pattern of expression was quantified. Both morphine- and oxycodone-exposed mice exhibited higher occludin fragmentation per square micrometer of microvessels. In addition, ZO-1 fragmentation was increased in oxycodone-exposed animals (Fig. 1*C,D*). These results indicate discontinuity of occludin and ZO-1 integrity after opioid exposure and correspond to decreased expression of these proteins as observed in Figure 1, *A* and *B*. On the other hand, claudin-5 fragmentation was significantly lower after both oxycodone and morphine exposure (Fig. 1*C,D*).

10.1523/JNEUROSCI.0614-24.2024.f1-1 Figure 1-1 **Chronic opioid exposure alters the brain and systemic inflammatory responses.** Supports Figure 1. Age-matched mice were chronically exposed to morphine or oxycodone as in Figure 1. **(A)** Expression levels of Iba-1 in the brains analyzed by immunoblotting. GAPDH was used as a loading control. The same GAPDH band is shown in Figure 1F since the same membrane was stained with different antibodies. **(B)** mRNA expression levels of CXCL1 and MMP9 in the brains as analyzed by qPCR. GAPDH was used as a housekeeping gene and loading control (n = 8 animals per group, 3 independent experiments). **(C)** Plasma levels of cytokines and chemokines measured by Multiplex Immunoassays (n = 15 animals per group, 3 independent experiments). Data are means ± SD. *p < 0.05, **p < 0.01, ***p < 0.001 or ****p < 0.0001. Download Figure 1-1, TIF file.

We then used an in vivo permeability assay to further evaluate whether the barrier function of the BBB was impacted by chronic opioid exposure. Compared to the control animals, both the morphine- and oxycodone-exposed mice were characterized by higher levels of sodium fluorescein in the brain tissue, indicating loss of BBB integrity (Fig. 1*E*), which was consistent with a decrease in expression and an increase in fragmentation of TJ proteins. Brain levels of fibrinogen β, a protein mainly distributed in the blood, can serve as another marker of BBB disruption (Petersen et al., 2018). Indeed, the level of fibrinogen β was also significantly increased in the brains of both morphine- and oxycodone-exposed mice, thereby confirming BBB disruption (Fig. 1*F*).

We confirmed these results in vitro in cultured primary human hBMEC. Cells were treated with morphine or oxycodone for 3 d, followed by a permeability assay with FITC-labeled dextran. The results indicated an increase in permeability of 4 and 10 kDa FITC-dextran was increased after the opioid treatment (Fig. 1*G*). In contrast, permeability of 20 and 40 kDa was not altered, indicating selective disruption of endothelial barrier function. Further confirming our in vivo data, immunoblotting results revealed that both occludin and ZO-1 were downregulated in opioid-treated cells, with no changes in claudin-5 expression levels (Fig. 1*H,I*). Overall, these results indicate that opioid exposure can selectively damage BBB integrity and that these effects can be reproduced in vitro.

In order to assess if opioid-mediated CNS injury is limited to microvasculature, we evaluated the level of the Iba-1, a marker of microglial activation (Jurga et al., 2020), and mRNA levels of inflammatory mediators CXCL1 and matrix metalloprotease (MMP) 9 by qPCR in brain homogenates of opioid-exposed mice. There was a significant increase in the Iba1 protein expression (Extended Data Fig. 1-1*A*) and mRNA levels of CXCL1 and MMP9 (Extended Data Fig. 1-1*B*) in the opioid-exposed mice as compared with the control group. CXCL1 has been involved in the recruitment of neutrophils and macrophages (Sawant et al., 2016), and MMP9 has been linked to the degradation of the extracellular matrix and regulation of neutrophil migration (Turner and Sharp, 2016).

We also assessed the global inflammation by measuring the cytokine plasma profile using a Bio-Plex Multiplex Immunoassay (Extended Data Fig. 1-1*C*). Elevated plasma levels of multiple proinflammatory cytokines, including IL-1α, IL-3, IL-6, TNF-α, CCL3, and CXCL1, were observed in opioid-exposed mice. In summary, these data demonstrated that chronic opioid exposure could induce both CNS and global inflammatory responses.

### Chronic opioid exposure results in enhanced superoxide generation in brain microvascular endothelium

**Figure 2.**
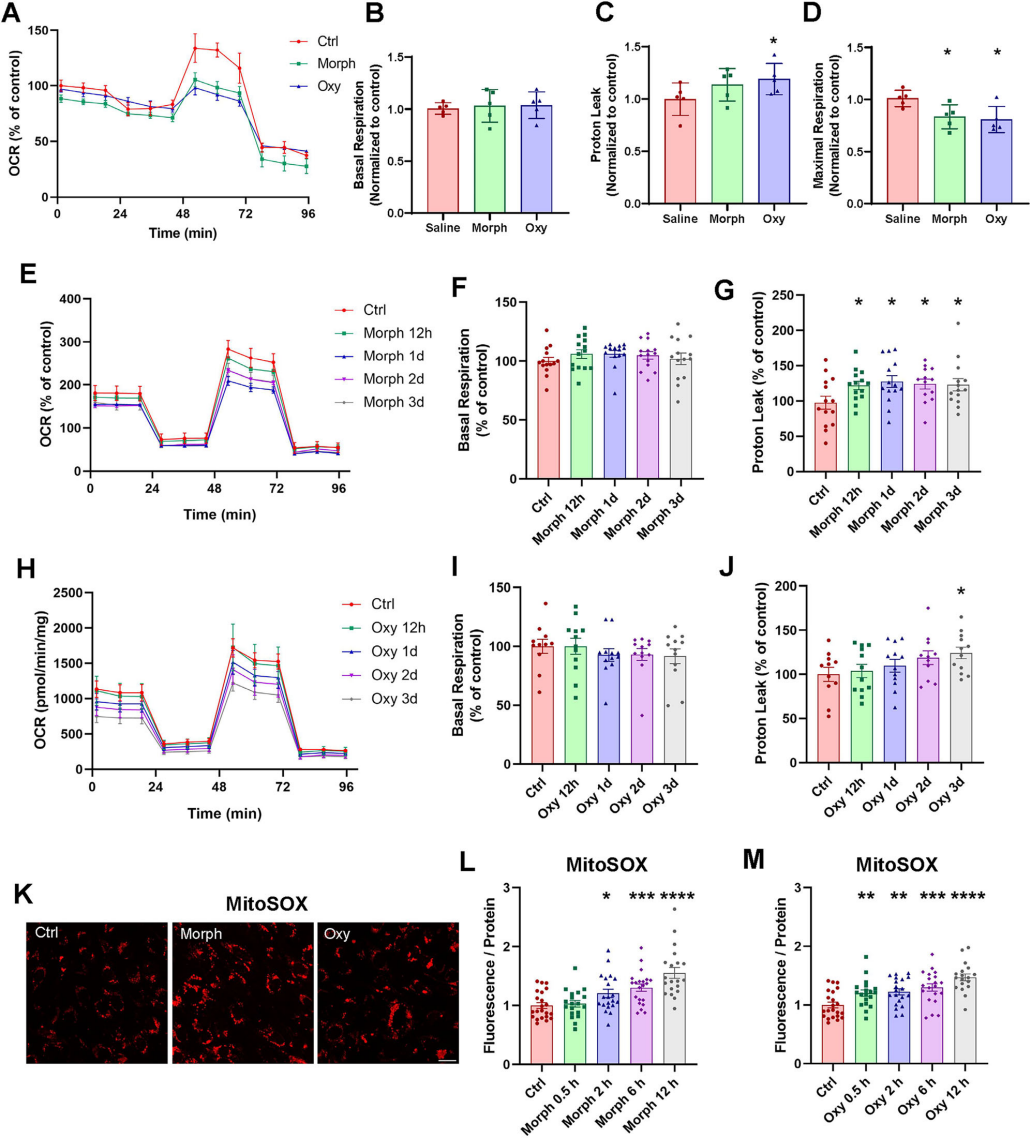
Endothelial mitochondrial damage and elevated ROS production induced by chronic opioid exposure. ***A–D***, Age-matched mice were chronically exposed to morphine or oxycodone as in Figure 1. ***A***, OCR curve measured by the mito stress test in brain microvessels isolated from opioid-exposed mice. ***B***, Basal respiration, (***C***) proton leak, and (***D***) ATP production quantified from the OCR test. ***A–D***, Data are means ± SD (*n* = 5 animals per group, 3 independent experiments). ***E–M***, hBMEC were treated with 50 μM morphine or oxycodone as in Figure 1. ***E***, ***H***, OCR curves measured by the mito stress test in hBMEC treated with morphine (***E***) or oxycodone (***H***). ***F***, ***I***, Basal respiration **(*F***, ***I*)** and proton leak (***G***, ***J***) quantified from the OCR test (*n* = 12 per group, 3 independent experiments). ***K***, Representative images of hBMEC treated with 50 μM morphine or oxycodone for 30 min and stained with MitoSOX red. Scale bar, 20 μm. ***L***, ***M***, Quantification of MitoSOX red fluorescence from hBMEC treated with morphine (***L***) or oxycodone (***M***) for the indicated time (*n* = 20 per group). Data are means ± SEM. *p < 0.05, **p < 0.01, ***p < 0.001, or ****p < 0.0001.

Both in vitro and in vivo studies have demonstrated that oxidative stress is involved in opioid exposure (Ward et al., 2020). Importantly, mitochondria serve as a major organelle in ROS generation and influence neurological functions (Alshial et al., 2023). Therefore, we evaluated whether opioid treatment could impact mitochondrial function in brain microvessels. The Seahorse mitochondrial stress test, a high-throughput technique to measure the respiratory rate, was used to evaluate the function of mitochondria in microvessels isolated from morphine- or oxycodone-treated mice. We observed a decreasing trend in the real-time oxygen consumption rate (OCR) in microvessels isolated from both morphine- and oxycodone-treated mice (Fig. 2*A*). While basic mitochondrial respiration was not altered by opioid exposure, there was a significant increase in proton leakage in the microvessels of oxycodone-treated mice, suggesting disruption to mitochondrial respiratory flux (Fig. 2*B,C*). Furthermore, there was a decrease in maximal mitochondrial respiration in microvessels of both morphine- and opioid-exposed mice, suggesting a diminished capacity to protect against tissue damage (Fig. 2*D*).

To further evaluate the impact of opioids on mitochondrial functions, brain endothelial cells were treated with opioids for different durations, and mitochondrial function was measured using the Seahorse mitochondrial stress test. OCR of human endothelial cells treated with morphine (Fig. 2*E*) exhibited a decreasing trend compared with the control group, which mimicked the results from brain microvessels of morphine-treated animals. Specifically, morphine treatment did not affect basal respiration (Fig. 2*F*); however, there was a significant increase in the proton leak, a marker of oxidative damage (Divakaruni and Brand, 2011), at all time points of morphine exposure (Fig. 2*G*). Similar results were obtained in brain endothelial cells exposed to oxycodone, except for the proton leak being observed only in cells treated with repeating doses of oxycodone for 3 d (Fig. 2*H–J*).

After confirming mitochondrial respiratory disruption, we measured the oxidative stress level in brain endothelial cells by evaluating the ROS production with MitoSOX red, a fluorescent superoxide probe (Fig. 2*K–M*). hBMEC treated with 50 μM morphine (Fig. 2*L*) or oxycodone (Fig. 2*M*) exhibited a statistically significant accumulation of superoxides. Together, these data support our hypothesis that elevated ROS production and mitochondrial oxidative damage in endothelial cells underly opioid-induced vascular toxicity.

### Chronic opioid exposure leads to mitochondria dysregulation and fragmentation in endothelial cells in vitro and in vivo

**Figure 3.**
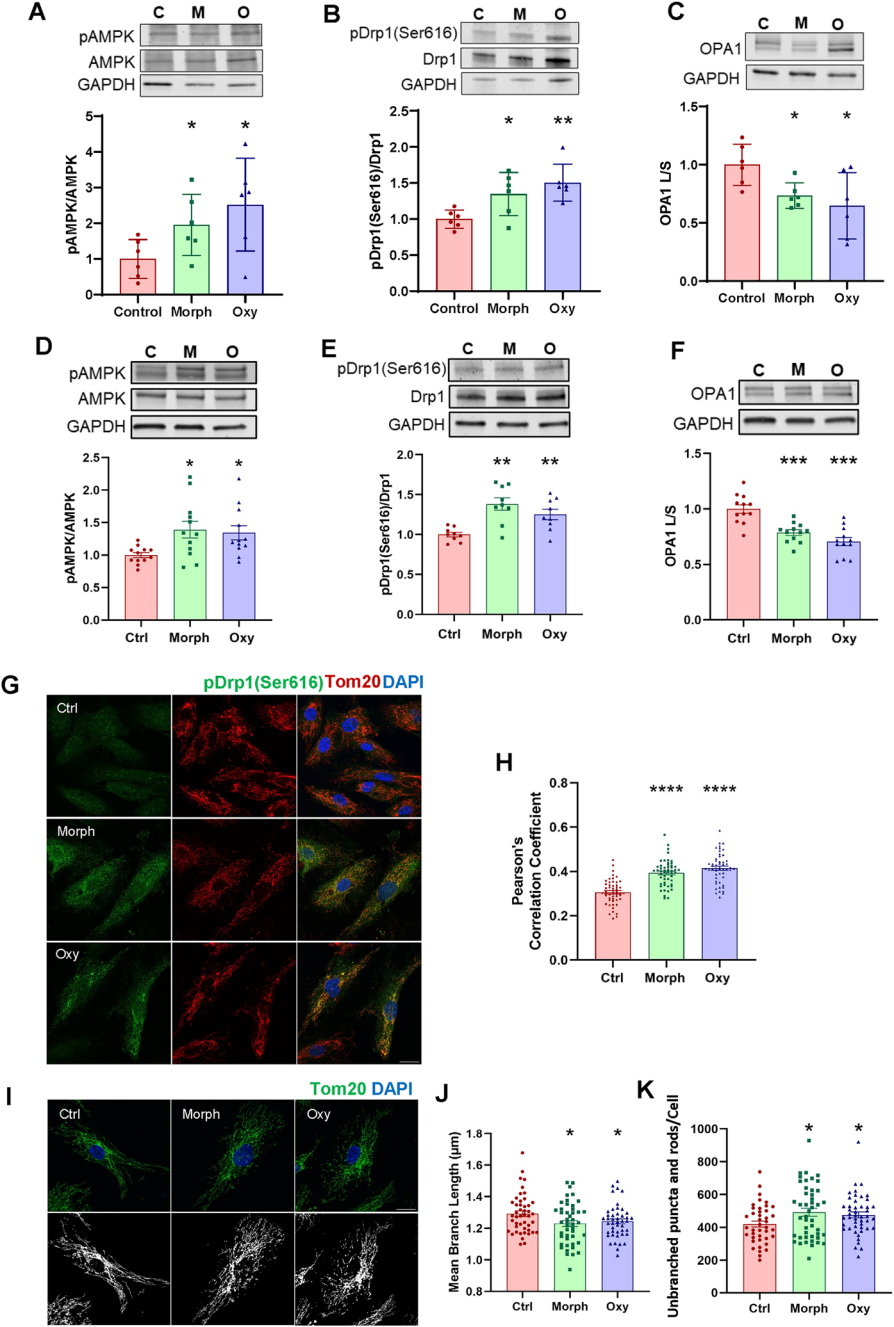
Chronic opioid exposure reprograms mitochondrial dynamics. ***A–C***, Age-matched mice were chronically exposed to morphine or oxycodone as in Figure 1. Phosphorylation of AMPK (***A***) and Drp1 (***B***) to their active forms and OPA1 expression (***C***) in brain microvessels from opioid-exposed mice as analyzed by immunoblotting. GAPDH was used as a loading control. ***A–C***, Data are means ± SD (*n* = 6 animals per group, 3 independent experiments). ***D–K***, hBMEC were treated with 50 μM morphine or oxycodone as in Figure 1. Phosphorylation of AMPK (***D***) and Drp1 (***E***) and the expression of OPA1 (***F***) as analyzed by immunoblotting. GAPDH was used as a loading control (*n* = 12 per group, 3 independent experiments). ***G***, Representative images of hBMEC stained for pDrp1(Ser616) (green.), TOM20 (red), and DAPI (blue). Scale bar, 20 μm. ***H***, Pearson's correlation coefficients between the fluorescence signal of pDrp1(Ser616) and TOM20 in hBMEC (*n* = 50 images per group). ***I***, Representative images of hBMEC stained with TOM20 (green) and DAPI (blue) and corresponding binarized images. Scale bar, 20 μm. ***J***, ***K***, The mean branch length (***J***) and the number of unbranched puncta and rods per cell (***K***) in the mitochondria network of hBMEC (*n* = 40 cells per group, 3 independent experiments). Data are means ± SEM. *p < 0.05, **p < 0.01, ***p < 0.001, or ****p < 0.0001.

In the next series of experiments, we further evaluated opioid-induced mitochondrial dysregulation in the brain microvasculature. Phosphorylation of AMP-activated protein kinase (AMPK), a major sensor of energy stress and mitochondrial insult (Herzig and Shaw, 2018), was elevated in brain microvessels isolated from morphine- or oxycodone-treated mice (Fig. 3*A*). AMPK can directly phosphorylate mitochondrial fission factor, the primary receptor for dynamin-like protein (Drp1) on the mitochondrial outer membrane (Archer, 2013), which then mediates the constriction of the mitochondrial membrane during normal fission processes. Consistent with activation of AMPK, phosphorylation of Drp1 at Ser616, which increases Drp1 activity (Horbay and Bilyy, 2016), was enhanced in microvessels of morphine- or oxycodone-treated mice (Fig. 3*B*). We also characterized the expression of the optic atrophy-1 (OPA1) protein in opioid-exposed mice. The long isoform of this protein mediates the fusion of the mitochondrial inner membrane; however, it can be cleaved into an inactive short isoform when the mitochondrial transmembrane potential is lost (Ge et al., 2020). As shown in Figure 3*C*, the ratio between the long and short isoforms of OPA1 was decreased in the opioid-treated cells, which would result in the inhibition of mitochondrial fusion (Gilkerson et al., 2021).

Enhanced phosphorylation of AMPK and Drp1 (Ser616; pDrp1), along with a decrease in the ratio of active to inactive isoforms of OPA1, which indicates the cleavage of the long isoforms of OPA1, were also observed in brain endothelial cells treated with morphine or oxycodone (Fig. 3*D–F*). These results are consistent with the mouse experiments, confirming unbalanced mitochondrial dynamics induced by chronic opioid exposure. Using immunostaining, we found that colocalization of pDrp1 with the mitochondrial marker TOM20 is significantly increased in brain endothelial cells treated with morphine or oxycodone, indicating recruitment of Drp1 to the mitochondria (Fig. 3*G,H*).

Both activation of Drp1 and increased cleavage of long OPA1 can enhance the mitochondrial fission process. Therefore, we analyzed the morphology of the mitochondrial network in opioid-treated hBMEC by using the MiNA toolset (Fig. 3*I*; Valente et al., 2017). The results revealed that mitochondria in opioid-treated cells had shorter mean branch lengths, which affect mitochondrial networks (Fig. 3*J*) and more unbranched puncta and rods per cell (Fig. 3*K*) as compared with controls, indicating higher fragmentation of the mitochondrial network. Overall, the results in Figure 3 demonstrate that treatment with morphine or oxycodone switched the balance of endothelial mitochondria dynamics from fusion to fission and induced extensive mitochondrial fragmentation.

### Chronic opioid exposure induces microvascular endothelial apoptosis

**Figure 4.**
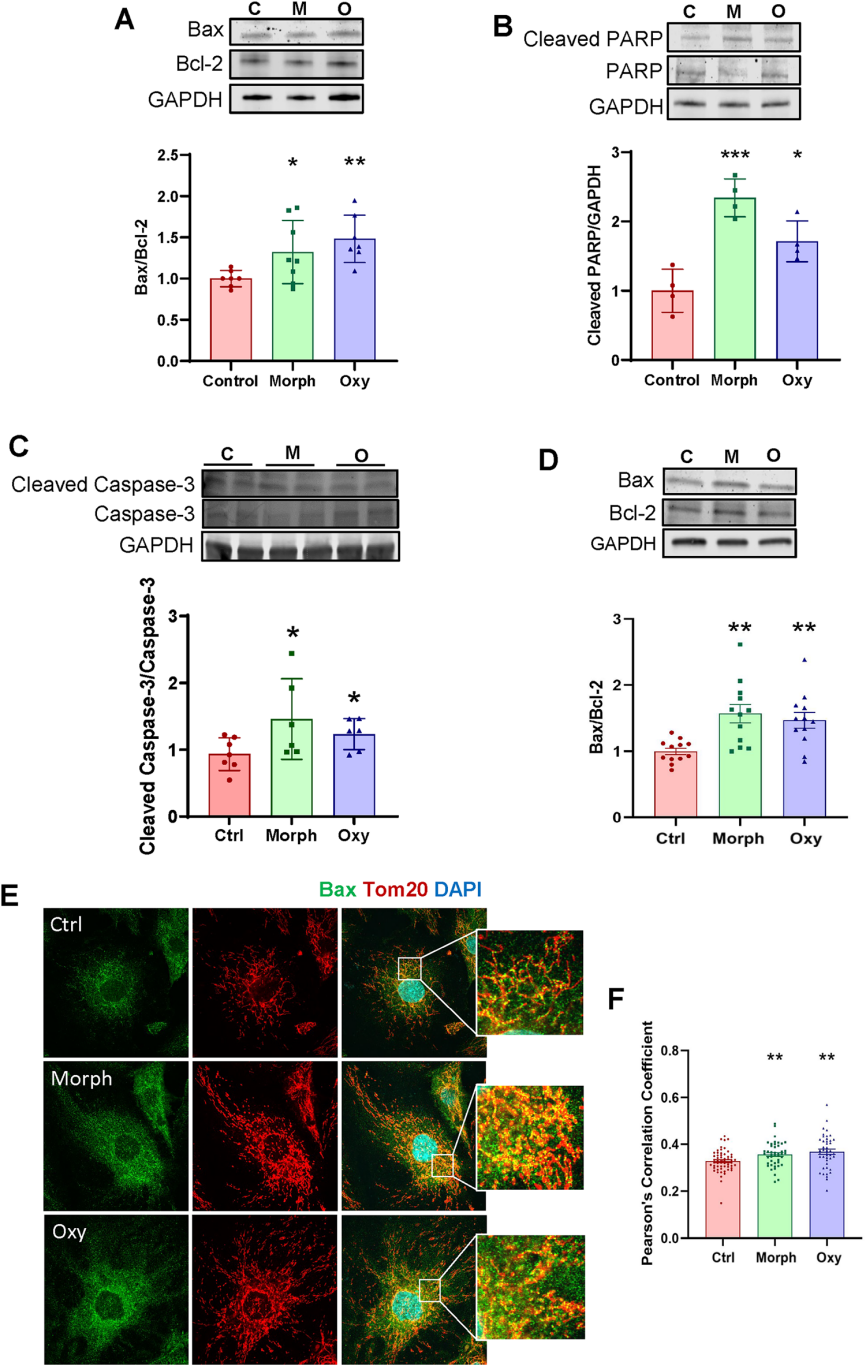
Chronic opioid exposure induces the expression of apoptotic genes in brain microvasculature. ***A–C***, Age-matched mice were chronically exposed to opioids as in Figure 1. ***A***, Expression of Bax and Bcl-2 and (***B***, ***C***) cleavage of PARP and caspase-3 in mouse brain microvessels as analyzed by immunoblotting. GAPDH was used as a loading control. ***A–C***, Data are means ± SD (*n* = 6 animals per group, 3 independent experiments). ***D–F***, hBMEC were treated with 50 μM morphine or oxycodone as in Figure 1. ***D***, Expression of Bax and Bcl-2 in opioid-treated hBMEC as analyzed by immunoblotting. GAPDH was used as a loading control (*n* = 12 per group, 3 independent experiments). ***E***, Representative images of hBMEC stained for Bax (green), TOM20 (red), and DAPI (blue). Scale bar, 20 μm. ***F***, Pearson's correlation coefficients between the fluorescence signals of Bax and TOM20 in hBMEC (*n* = 50 images per group). Data are means ± SEM. *p < 0.05, **p < 0.01, ***p < 0.001, or ****p < 0.0001.

Since elevated oxidative stress and mitochondrial dysfunction can be associated with apoptosis (Redza-Dutordoir and Averill-Bates, 2016), which has been linked to ischemic stroke (Tuo et al., 2022), we further evaluated apoptosis markers in brain microvessels isolated from morphine or oxycodone-exposed mice. The mitochondrial pathway of apoptosis is regulated by the balance between Bax and Bcl-2, both members of the Bcl-2 family, where changes in the ratio between these two proteins can influence and determine cell fate (Redza-Dutordoir and Averill-Bates, 2016; Kale et al., 2018). Bax exists in an inactive form in the cytosol of healthy cells. In response to apoptotic stimuli, Bax is inserted into the outer mitochondrial membrane where it is oligomerized and wields its proapoptotic function (Pawlowski and Kraft, 2000; Kale et al., 2018). As a result, cytochrome *c* is released from the mitochondria to the cytosol, which activates the caspase cascades. Our results provide evidence that the Bax/Bcl-2 ratio is elevated in microvessels of morphine- or oxycodone-treated mice (Fig. 4*A*), indicating a reprogramming toward the apoptosis pathway. Furthermore, the cleavage of poly-(ADP-ribose) polymerase (PARP), a product of activated caspases (Pawlowski and Kraft, 2000), and the cleavage of caspase-3 were also increased in the microvessels of opioid-treated mice (Fig. 4*B,C*).

The Bax/Bcl-2 ratio was also analyzed by immunoblotting in cultured brain endothelial cells exposed to morphine or oxycodone. Consistent with the results in microvessels, we detected a significantly higher Bax/Bcl-2 ratio in cells treated with opioids, indicating their proapoptotic fate (Fig. 4*D*). We also detected a translocation of Bax protein into mitochondria. Indeed, either morphine or oxycodone treatment increased colocalization between Bax and TOM20 (Fig. 4*E,F*). In summary, these results indicate that chronic opioid exposure stimulates proapoptotic pathways in microvascular endothelium.

### NAC attenuates ischemic stroke damage after chronic opioid exposure

Opioid dependency is a risk factor for ischemic stroke, and microvascular endothelial dysfunction observed in postopioid exposure may impact the severity of ischemic stroke. Therefore, we evaluated the impact of chronic morphine and oxycodone exposure on stroke outcomes. Because of the strong oxidative component of opioid-induced microvascular toxicity, we introduced an antioxidant and anti-inflammatory mediator, *N*-acetylcysteine (NAC). Indeed, treatment with NAC was reported to improve neurological outcomes in poststroke patients (Sabetghadam et al., 2020).

**Figure 5.**
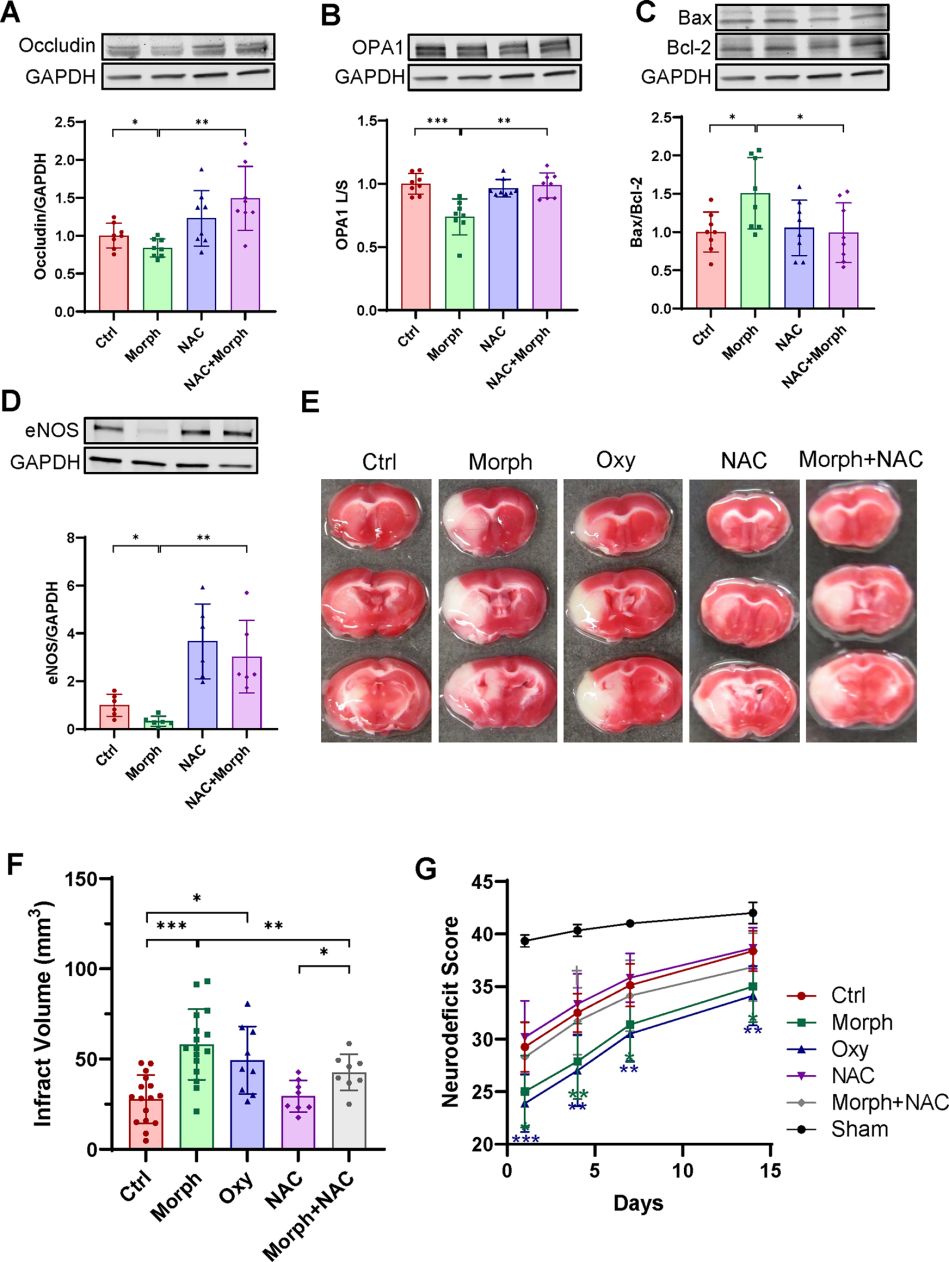
NAC treatment attenuates the outcome of ischemic stroke and accelerates poststroke recovery in mice chronically exposed to opioids. ***A–D***, Age-matched mice were chronically exposed to morphine as in Figure 1 and treated with 150 mg/kg body weight of NAC for the last 5 d of exposure. Expression of occludin (***A***), OPA1 (***B***), Bax/Bcl-2 (***C***), and eNOS (***D***) in mouse brain microvessels as analyzed by immunoblotting. GAPDH was used as a loading control. The same GAPDH band is shown in ***A***, ***B***, and ***C*** since the same membrane was stained with different antibodies. ***A–D***, Data are means ± SD (*n* = 6–8 animals per group, 3 independent experiments). ***E–G***, Mice were chronically exposed to morphine or oxycodone for 14 d, treated with 150 mg/kg body weight of NAC for the last 5 d, and subjected to ischemic stroke by the MCAO procedure 1 d after the last dose of opioid administration and NAC treatment. The brains were harvested 24 h post reoxygenation, sectioned into 1 mm slices, and stained with TTC (*n* = 6–9 animals per group, 3 independent experiments). ***E***, TTC staining of brain sections of mice after 24 h of stroke. ***F***, Infarct volume quantification. ***G***, Neurodeficit scores post-MCAO mice evaluated on Days 1, 4, 7, and 14 poststroke. ***E–G***, Data are means ± SD, *n* = 6–16 animals per group, 3 independent experiments). *p < 0.05, **p < 0.01, ***p < 0.001, or ****p < 0.0001.

Using immunoblotting, we first evaluated if NAC could ameliorate opioid-induced BBB damage, mitochondrial dysfunction, or apoptosis. NAC exposure elevated occludin levels and protected against mitochondrial damage, as assayed by the ratio of the long to short OPA isoforms, and apoptosis, as measured by the Bax/Bcl-2 ratio, in brain microvessels isolated from morphine-treated mice (Fig. 5*A–C*). Levels of the endothelial nitric oxide synthase (eNOS), a central regulator of endothelial homeostasis (Heiss et al., 2015), were examined in microvessels and found to be significantly downregulated by morphine exposure. In addition, these effects were also significantly attenuated by NAC treatment (Fig. 5*D*).

Next, we evaluated whether chronic opioid exposure could contribute to ischemic stroke outcomes and if NAC treatment may improve stroke deficits. Mice were exposed to morphine or oxycodone for 14 d and cotreated with NAC (or vehicle, PBS) for the last 5 d. Then, ischemic stroke was induced by the MCAO method. Both morphine and oxycodone exposure significantly enhanced the size of infarct volume when compared with control mice. Importantly, NAC significantly protected against this effect both in the control and opioid-exposed mice (Fig. 5*E,F*). In addition, NAC improved motor functions and poststroke recovery as assessed by the neurodeficit score (Fig. 5*G*). This effect was observed already 24 h poststroke, corresponding to acute poststroke lesion, and was maintained throughout poststroke recovery (i.e., poststroke days 7 and 14).

### NLRP3 inflammasome activation worsens ischemic stroke outcome after chronic opioid exposure

**Figure 6.**
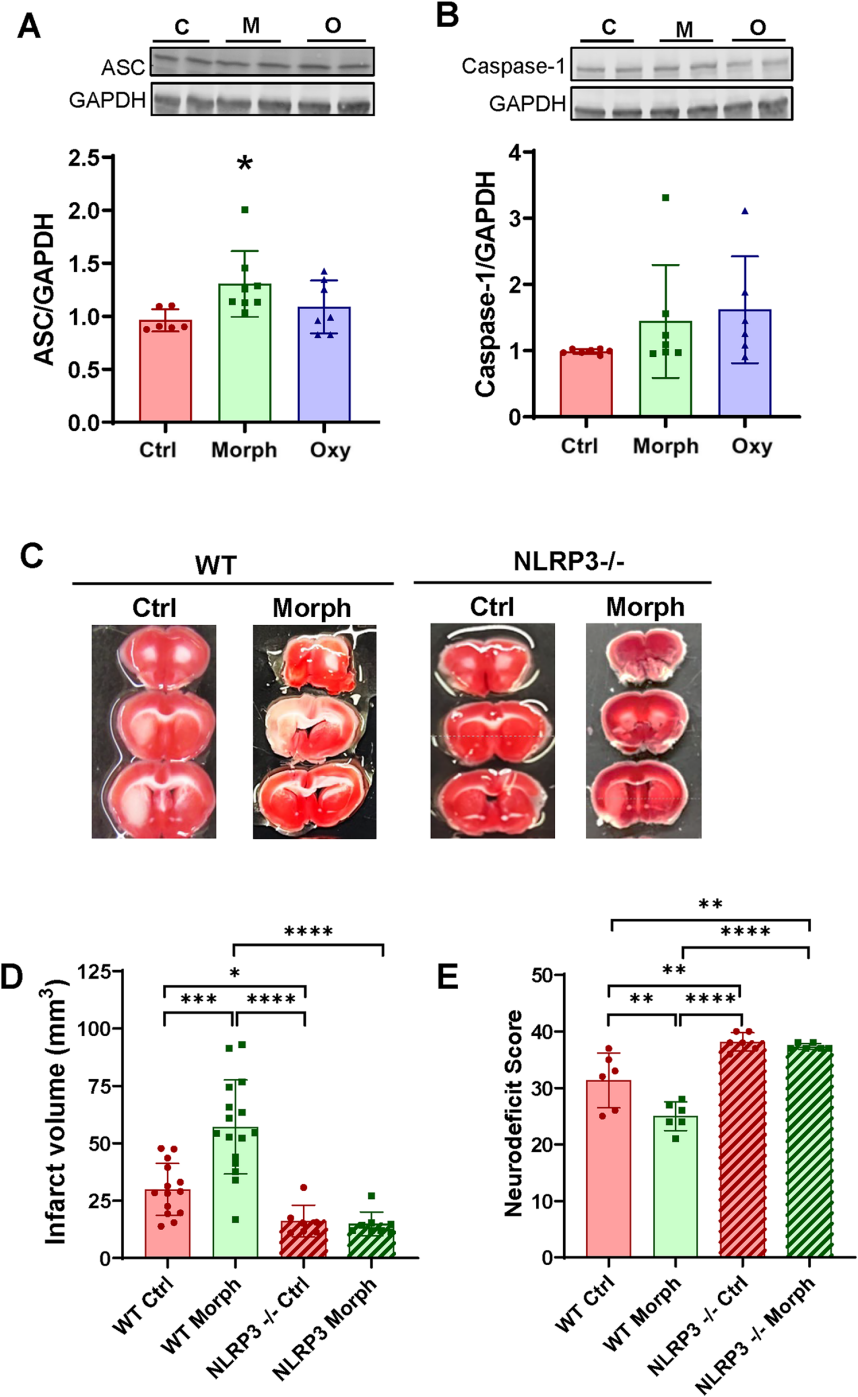
The NLRP3 inflammasome is involved in chronic opioid-induced potentiation of ischemic stroke. ***A***, ***B***, Age-matched wild-type mice were chronically exposed to morphine or oxycodone as in Figure 1. Protein expression of ASC (***A***) and caspase-1 (***B***) was analyzed in mouse brain microvessels by immunoblotting. GAPDH was used as a loading control. ***C–E***, Wild-type and the NLRP3 inflammasome-deficient mice were exposed to morphine as in ***A*** and ***B***, followed by ischemic stroke by the MCAO procedure 1 d after the last dose of opioid administration. The brains were harvested 24 h post reoxygenation and sectioned into 1 mm slices and stained with TTC (*n* = 6 animals per group, 3 independent experiments). ***C***, Representative TTC staining of brain sections of mice 24 h poststroke. ***D***, Infarct volume quantification. ***E***, Neurodeficit scores evaluated 24 h poststroke. ***A–E***, Data are means ± SD (*n* = 6–16 animals per group, 3 independent experiments). *p < 0.05, **p < 0.01, ***p < 0.001, or ****p < 0.0001.

Several studies have shown that the relation between the use of opioids and the inflammasome activation (Cui et al., 2021; Zare et al., 2023) and activated NLRP3 inflammasome may affect the severity of a stroke (Torices et al., 2023). The NLRP3 inflammasome is constructed by the NLRP3 protein engaging with the adapter protein ASC and the inflammatory pro-caspase-1. Therefore, we evaluated the expression levels of ASC and caspase-1 in brain microvessels isolated from morphine- and oxycodone-exposed mice. ASC is an integral protein for the inflammasome, working as the central adaptor for the complex. Caspase-1 is one of the effector proteins of the inflammasome pathway. There was a significant increase in the ASC expression in microvessels isolated from morphine-exposed mice and a strong tendency to enhance caspase-1 levels in both morphine- and oxycodone-treated mice (Fig. 6*A,B*).

We then evaluated ischemic stroke outcomes in the NLRP3-deficient mice. Mice were exposed to morphine, and an ischemic stroke was induced by the MCAO procedure for 1 h followed by reperfusion for 24 h. Consistent with the results from Figure 5, treatment with morphine enhanced poststroke lesion volume. Most interestingly, the NLRP3-deficient mice were fully protected against the development of ischemic lesions. The infarct volume was also significantly lower in morphine-treated NLRP3-deficient mice when compared with morphine-treated wild-type mice (Fig. 6*C,D*). Consistent with these results, the NLRP3-deficient mice also presented excellent poststroke motor function as assessed by NeuroScore (Fig. 6*E*).

## Discussion

In clinical practices, opioid therapy is titrated and begins with the lowest effective dosage (Dowell et al., 2016), which is then increased to account for the developing tolerance. A similar escalation of opioid uptake occurs during opioid misuse disorder. The elimination half-life of morphine and oxycodone, two common prescription opioids, in the plasma of humans is ∼4 h (De Gregori et al., 2014), while the half-life in mice is ∼0.5 h (Dalesio et al., 2016). In the present study, we used a daily escalating dose regimen twice per day with a 6 h interval for 2 weeks. Based on the extrapolation from the clinical dosages in humans (Nair and Jacob, 2016), the stepwise increasing dose ranged for morphine from 1 to 15 mg/kg and for oxycodone from 0.6 to 10 mg/kg.

While studies have investigated endothelial toxicity of opioids (Marino et al., 2022; Reymond et al., 2022), the conclusions are controversial as most of the literature reports in vitro evidence with a single opioid dosage, which limits the translational relevance of findings. Several groups have shown that opioids, particularly morphine, may disrupt the expression of TJ proteins, resulting in decreased endothelial integrity (Leibrand et al., 2019; Peyravian et al., 2022). Others have reported that morphine exposure may not play any significant role in endothelial injury in cell culture (Marino et al., 2022) and in rats (Yousif et al., 2008). Here, we provide both in vivo and in vitro evidence that chronic opioid exposure induces structural and functional alterations of the brain vascular endothelium, such as a decrease in the expression of the major TJ proteins and an increase in BBB permeability (Fig. 1).

We also sought to determine whether chronic opioid exposure could elevate oxidative stress, which could contribute to the disruption of BBB (Feng et al., 2023). Our experiments revealed an enhancement in ROS production and oxidative damage in brain microvessels isolated from opioid-exposed mice and human hBMEC cells treated with opioids (Fig. 2). These findings are in line with studies showing the association between opioid dependence and oxidative stress (Ward et al., 2020; Tobore, 2021).

It has been shown that aberrant changes in mitochondrial dynamics are a common feature in numerous neurodegenerative diseases, whereby an imbalance in mitochondrial division, fusion, or mitophagy can enhance neurological pathology (D. Yang et al., 2021). Therefore, we asked if chronic opioid exposure could alter the functions and the structure of mitochondrial network. Indeed, chronic opioid treatment switched the balance of mitochondrial dynamics from fusion to fission and induced mitochondrial fragmentation. We also observed activation of AMPK, a key sensor of mitochondria insult (Herzig and Shaw, 2018), and an increase in Drp1 Ser616 phosphorylation, which mediates constriction of the mitochondrial membrane during fission (Archer, 2013). Consistently, the ratio between long and short isoforms of OPA1, a regulator of mitochondrial fusion (Gilkerson et al., 2021), decreased with opioid exposure. Together, these results elucidate an increase in mitochondrial fission in microvessels, which was consistent with a higher fragmentation of the mitochondrial network following opioid treatment (Fig. 3). These results are in line with the notion that changes in mitochondrial dynamics have been associated with alterations in mitochondrial morphology (Westrate et al., 2014).

Our extended results provide evidence that the toxic impact of chronic opioid exposure is not limited to the brain microvasculature but can also lead to microglia activation and increase systemic inflammation. In the brain of opioid-exposed mice, we indicated an increase in the expression of Iba-1, CXCL1, and MMP9, the proinflammatory factors that are involved in the recruitment and transmigration of proinflammatory cells into the brain (Turner and Sharp, 2016). These observations are consistent with the evidence that morphine exposure can induce neuroinflammation (Wang et al., 2012; Eidson et al., 2017) and lead to increased cell migration into the CNS (Strazza et al., 2016). Furthermore, chronic exposure to opioids resulted in an increase in plasma levels of multiple proinflammatory cytokines, including IL-1α, IL-3, IL-6, TNF-α, CCL3, and CXCL1 (Extended Data Fig. 1-1).

Dysregulation in mitochondrial dynamics has been linked to apoptosis (Redza-Dutordoir and Averill-Bates, 2016), so we hypothesized that opioid-induced mitochondrial dysfunction could further activate the apoptotic cascade, which could contribute to the loss of TJ proteins. Indeed, mitochondria are central mediators of apoptosis, which is initiated by cellular stressors, including ROS and mitochondrial fission that involves DRP1 and the Bax/Bak pathway (Giorgio et al., 2005; Suen et al., 2008). These pathways are closely connected as mitochondrial ROS have been shown to activate p53, which leads to Bax/Bak translocation and release of cytochrome *c* to initiate the apoptosis cascade (Cheng et al., 2007). Therefore, it was important that chronic opioid exposure resulted in an increase in the Bax/Bcl-2 ratio and PARP cleavage (Fig. 4). The Bax/Bcl-2 ratio is a key regulator of cell apoptosis. Once activated, Bax is inserted into the outer mitochondrial membrane where it activates the caspase cascade through PARP (Pawlowski and Kraft, 2000; Kale et al., 2018). These alterations are in line with previous studies demonstrating that morphine can induce apoptosis in human endothelial cells through the ROS pathways (Hsiao et al., 2009). Furthermore, our results are in agreement with studies using chronic opioid exposure in vitro, where it was described that morphine can induce microglial immunosuppression via activation of insufficient mitophagy (Peng et al., 2022) as well as in vivo in mice (Murlanova et al., 2022).

Increased BBB permeability results in neuroinflammation, one of the major contributors to vascular damage and mortality in ischemic stroke (Li et al., 2023). Furthermore, studies have shown a direct relationship between mitochondrial disruption (Doll et al., 2015, Shen et al., 2021), apoptosis (X. Liu et al., 2023), and ischemic stroke damage and recovery. Our results indicated that chronic opioid exposure can lead to BBB disruption, mitochondrial dysfunction, and apoptosis, i.e., the processes that can directly influence the severity of ischemic stroke damage. Therefore, we next evaluated the impact of chronic opioid exposure on the outcome of ischemic stroke. We observed significantly larger tissue damage and diminished functional recovery in opioid-exposed mice post MCAO. In fact, the animals exposed to morphine almost doubled ischemic infarct lesions compared with the controls (Fig. 5). These results are in agreement with reports on acute exposure to opioids showing a relation between increased ischemic stroke damage and opioid abuse (Peyravian et al., 2022).

Due to its role as an antioxidant and anti-inflammation agent, the use of NAC has been reported to improve neurological outcomes in poststroke patients (Sabetghadam et al., 2020; Komakula et al., 2024). Our novel results indicate significantly higher levels of occludin after NAC supplementation, combined with attenuated mitochondrial fission and apoptosis as demonstrated by diminished OPA1 levels and a decrease in the Bax/Bcl-2 ratio. Most importantly, stroke damage and poststroke recovery were significantly improved when mice were supplemented with NAC during the last 5 d of opioid exposure (Fig. 5). These results are consistent with reports showing that compromised BBB and elevated neuroinflammation contribute to the development of stroke (Obermeier et al., 2013).

Studies have reported a relationship between the use of opioids and inflammasome activation (Zare et al., 2023). Therefore, we hypothesized that the NLRP3 inflammasome could play a key role in stroke damage. Once activated, NLRP3 interacts with the adaptor protein ASC to recruit and activate caspase-1 which leads to the release of IL-1β and initiation of the inflammatory responses (Torices et al., 2023). Several studies have related BBB damage to NLRP3 activation in ischemic stroke showing that NLRP3 inhibition can reduce infarct size and improve endothelial cell survival and BBB integrity (F. Yang et al., 2014; Cao et al., 2016). In line with our results, studies have also shown a correlation between NLRP3 activation and potassium efflux, the release of mitochondrial reactive oxygen species, and lysosomal damage (Tong et al., 2015; Minutoli et al., 2016). Moreover, it has been shown that Nrf2 can inhibit the ROS-mediated activation of the NLRP3 inflammasome after oxygen–glucose deprivation in microglial cells (Xu et al., 2018) and that NLRP3 can be activated by oxidized mitochondrial DNA (Shimada et al., 2012). Interestingly, oxidative stress plays a vital role in the pathophysiology of ischemic stroke (Rodrigo et al., 2013). In the present study, we demonstrate an increase in the inflammasome ASC and caspase-1 in brain microvessels of morphine-exposed mice, combined with remarkable protection against the development of ischemic lesions in the NLRP3-deficient mice (Fig. 6). These results suggest that the NLRP3 inflammasome should be considered a new treatment target in ischemic stroke and stroke recovery, with or without concomitant exposure to opioids.

In summary, this study provides the first mechanistic insight into the correlation between chronic opioid exposure and ischemic stroke outcomes. We suggest that opioid-mediated cerebrovascular dysfunction due to mitochondrial dysfunction, elevation of ROS production, activation of inflammasomes, and apoptosis are critical factors that contribute to opioid-mediated potentiation of ischemic lesions and delayed poststroke recovery. Importantly, inhibition of the NLRP3 inflammasome or supplementation with NAC attenuated the opioid-induced cerebrovascular dysfunction and ischemic stroke lesions, as well as enhanced poststroke recovery, indicating the effectiveness of a targeted anti-inflammatory intervention for stroke treatment.
