# Supplementary figures and images for: Microvascular Dysfunction, Mitochondrial Reprogramming, and Inflammasome Activation as Critical Regulators of Ischemic Stroke Severity Induced by Chronic Exposure to Prescription Opioids
Source: J Neurosci. 2025 Jan 3;45(8):e0614242024. doi: 10.1523/JNEUROSCI.0614-24.2024 (PMC11841762; doi:10.1523/JNEUROSCI.0614-24.2024)

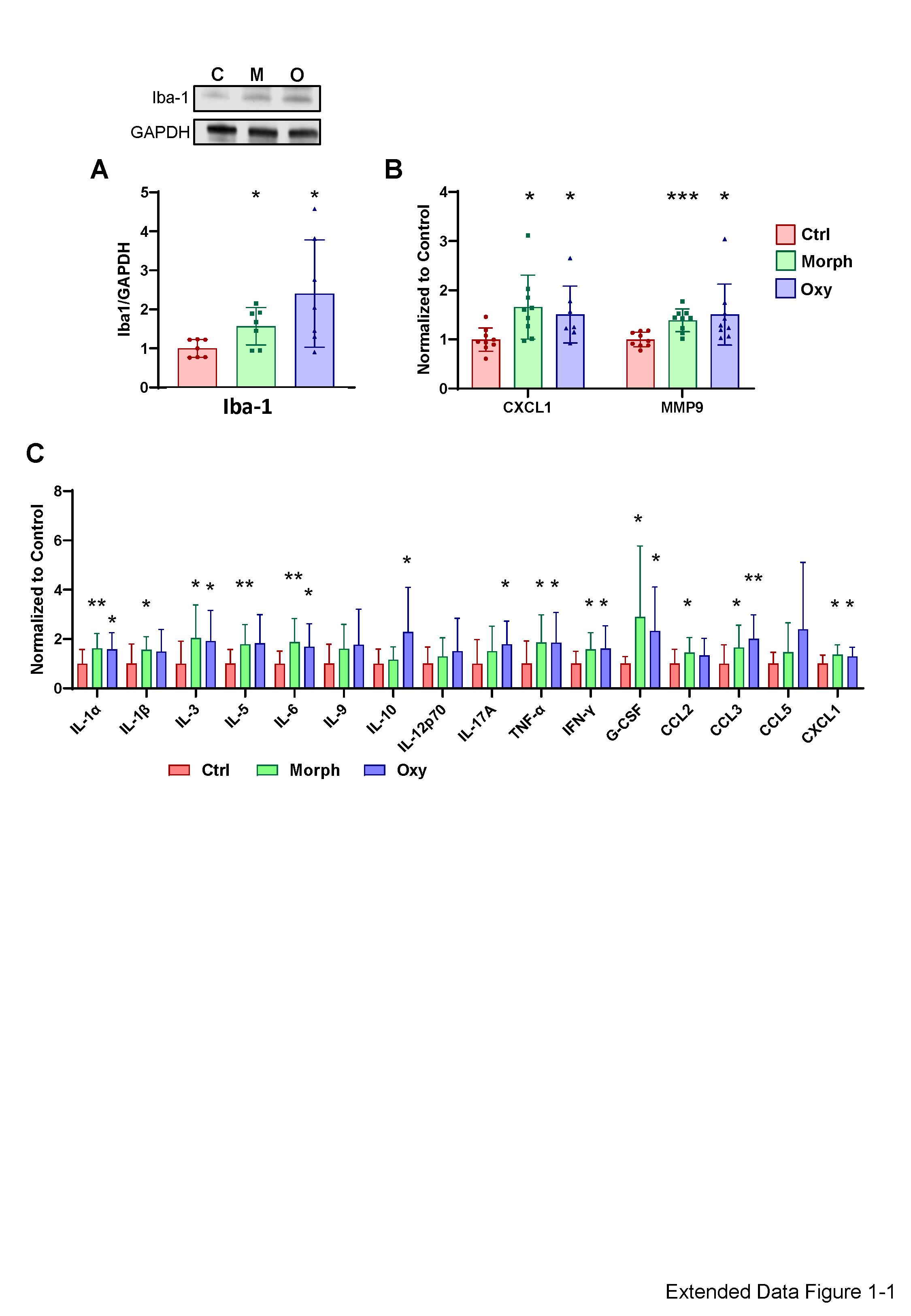

Supplement: Figure 1-1 — Chronic opioid exposure alters the brain and systemic inflammatory responses. Supports Figure 1. Age-matched mice were chronically exposed to morphine or oxycodone as in Figure 1. (A) Expression levels of Iba-1 in the brains analyzed by immunoblotting. GAPDH was used as a loading control. The same GAPDH band is shown in Figure 1F since the same membrane was stained with different antibodies. (B) mRNA expression levels of CXCL1 and MMP9 in the brains as analyzed by qPCR. GAPDH was used as a housekeeping gene and loading control (n = 8 animals per group, 3 independent experiments). (C) Plasma levels of cytokines and chemokines measured by Multiplex Immunoassays (n = 15 animals per group, 3 independent experiments). Data are means ± SD. *p < 0.05, **p < 0.01, ***p < 0.001 or ****p < 0.0001. Download Figure 1-1, TIF file. [file jneuro-45-e0614242024-s001.tif]
